# The associations between domain-specific sedentary behaviours and dietary habits in European adults: a cross-sectional analysis of the SPOTLIGHT survey

**DOI:** 10.1186/s12889-016-3708-3

**Published:** 2016-10-06

**Authors:** Sofie Compernolle, Katrien De Cocker, Pedro J. Teixeira, Jean-Michel Oppert, Célina Roda, Joreintje D. Mackenbach, Jeroen Lakerveld, Martin McKee, Ketevan Glonti, Harry Rutter, Helga Bardos, Greet Cardon, Ilse De Bourdeaudhuij, on behalf of the WP3 SPOTLIGHT group

**Affiliations:** 1 Department of Movement and Sport Sciences, Faculty of Medicine and Health Sciences, Ghent University, Watersportlaan 2, Ghent, B-9000 Belgium; 2 Research Foundation Flanders (FWO), Brussels, Belgium; 3 Interdisciplinary Center for the Study of Human Performance, Faculty of Human Kinetics, University of Lisbon, Lisbon, Portugal; 4 Equipe de Recherche en Epidémiologie Nutritionnelle (EREN), Centre de Recherche en Epidémiologie et Statistiques, Inserm (U1153), Inra (U1125), Cnam, COMUE Sorbonne Paris Cité, Université Paris 13, F-93017 Bobigny, France; 5 Department of Nutrition Pitié-Salpêtrière Hospital (AP-HP), Centre for Research on Human Nutrition Ile-de-France (CRNH IdF), Institute of Cardiometabolism and Nutrition (ICAN), Université Pierre et Marie Curie-Paris 6, Paris, France; 6 Department of Epidemiology & Biostatistics, EMGO Institute for Health and Care Research, VU University Medical Center, Amsterdam, The Netherlands; 7 ECOHOST-The Centre for Health and Social Change, London School of Hygiene and Tropical Medicine, London, UK; 8 Department of Preventive Medicine, Faculty of Public Health, University of Debrecen, Debrecen, Hungary

**Keywords:** Sitting time, Eating behaviour, Obesity

## Abstract

**Background:**

Sedentary behaviour has been associated with obesity and related chronic diseases. Disentangling the nature of this association is complicated due to interactions with other lifestyle factors, such as dietary habits, yet limited research has investigated the relation between domain-specific sedentary behaviours and dietary habits in adults. The aim of this paper was to examine the association between domain-specific sedentary behaviours and dietary habits in adults and to test the moderating effect of age and gender on this association.

**Methods:**

A total of 6,037 participants from five urban regions in Europe completed an online survey, of which 6,001 were included in the analyses. Multilevel mixed-effects logistic regression analyses were used to examine main associations and interaction effects.

**Results:**

All domain-specific sedentary behaviours, except transport-related sitting time, were significantly related to dietary habits. In general, having a higher sitting time was related to having less healthy dietary habits, especially for television viewing. Gender did not moderate any of the relations, and age was only a significant moderator in the relation between other leisure sitting time and alcohol consumption.

**Conclusion:**

Domain-specific sitting behaviours were related to unhealthy dietary behaviours. However, the small effect sizes suggest that individual level behavioural interventions focusing on sedentary behaviour will not be sufficient to improve dietary habits. The fact that almost none of the associations were moderated by age or gender suggests that these associations, and possibly also the effects of interventions targeting both behaviours, may hold across age and gender groups.

## Background

Sedentary behaviour, defined as any waking activity characterized by an energy expenditure of ≤ 1.5 metabolic equivalents and a sitting or reclining posture [1], has increased considerably in countries at all levels of development [2–4]. This increase is mainly attributed to changes in transport, entertainment and workplace environments, and has been linked to increases in obesity and related chronic diseases [5, 6]. Disentangling the nature of this association is complicated by interactions with other lifestyle factors, such as dietary habits [7, 8]. In particular, it is hypothesized that the association between television viewing and obesity may be due to the increased snacking behaviour associated with television viewing, rather than the action of sitting in front of the television [7].

This hypothesis is derived from evidence for an association between television viewing and unhealthy dietary habits [7, 9, 10]. However, the available evidence is strong for children and adolescents, but limited for adults [7, 9]. Moreover, previous studies focused solely on television viewing, only one domain of sedentary behaviour. Other domains, including transport-related, work-related, and other leisure-time sedentary behaviour [11], have not to date been associated with dietary habits. Nevertheless, there is a need to gain insight into any potential associations between these domain-specific sedentary behaviours and dietary habits, as many adults spend a substantial amount of time sitting in contexts other than domestic television viewing [12–14].

Additionally, it may be that the association between television time and dietary habits does not apply to everyone. Research by Pearson et al. [7] suggests that two demographic characteristics may moderate the association, namely age and gender. However, here too, evidence in adults is limited [7].

To address these gaps in the evidence, the first aim of this study was to explore associations between domain-specific sedentary behaviours and dietary habits in adults. Second, it assessed whether any association differed by age and gender. A better understanding of these associations could help guide future health promotion interventions.

## Methods

### Study design and sampling

This study was part of the SPOTLIGHT project [15], which was established to increase and combine knowledge on the wide range of overweight and obesity-related determinants to support effective health promotion approaches. The study was conducted in five urban regions in Belgium, France, Hungary, the Netherlands and the United Kingdom. Sampling of neighbourhoods and recruitment of participants has been described in detail elsewhere [16]. Briefly, neighbourhood sampling was based on a combination of residential density and socioeconomic status (SES) data at the neighbourhood level. This resulted in four types of neighbourhoods: low SES/ low residential density, low SES/ high residential density, high SES/ low residential density and high SES/ high residential density. In each urban region, three neighbourhoods of each neighbourhood type were randomly sampled (ie 12 neighbourhoods per urban region, 60 neighbourhoods in total). The aim was to recruit at least 100 participants per neighbourhood (6,000 in total) with an anticipated response rate of around 10 %. As we expected lower response rates from participants in low SES neighbourhoods [17], we oversampled adults (≥18 years) from low SES neighbourhoods (1200 adults per neighbourhood) relative to high SES neighbourhoods (800 adults per neighbourhood). Subsequently, this random sample of adult (≥18 years) inhabitants was invited by letter to participate in an online survey. This letter included general information of the study (eg the purpose, duration, confidentiality and voluntary nature), as well as a personal respondent number. By entering this respondent number on the website, participants were directed to the online survey. On the first page of this online survey, general study information was repeated briefly, followed by an informed consent. After providing informed consent, participants could fill out the online survey, containing questions on demographics, neighbourhood perceptions, social environmental factors, health, motivations and barriers for healthy behaviour, obesity-related behaviours and weight and height. The study was approved by the corresponding local ethics committees of participating countries.

### Measures

#### Sedentary behaviours

Domain-specific sedentary behaviours were assessed using the Marshall questionnaire [18]. This questionnaire evaluated the average time spent sedentary while travelling, working, watching television, using a computer and doing other leisure time activities on both weekdays and weekend days by asking the following question: “During the last seven days, please estimate how much time you usually spent sitting in each of the following activities on a weekday and a weekend day [18].” Subsequently, the five different domains were listed, and participants were asked to provide an estimate of the average time spent sitting per domain on a usual weekday/weekend day. Total sedentary time per domain was estimated by summing the weekday (multiplied by five) and weekend day (multiplied by two) minutes. The sum (minutes/week) was divided by 420 (as there are seven days per week, and 60 minutes per hour) to express mean domain-specific sedentary time in hours/day. Marshall. et al. showed that the questionnaire has acceptable criterion validity, with highest validity coefficients found for sitting time at work and using a computer at home (Pearson correlation *r* = 0.69–0.74) [18].

#### Dietary habits

Dietary habits were measured using single items from food frequency questionnaires by asking: ‘How many times a week do you usually eat/drink 1) fruit, 2) vegetables, 3) sugar-sweetened beverages, 4) alcoholic beverages, 5) sweets and 6) fast food?’. Response options were coded as follows: 0.5 = ‘once a week or less’, 2 = ‘2 times a week’, 3 = ‘3 times a week’, 4 = ‘4 times a week’, 5 = ‘5 times a week’, 6 = ‘6 times a week’, 7 = ‘7 times a week’, 14 = ‘twice a day’, and 21 = ‘more than twice a day’.

#### Socio-demographic variables, BMI and moderate-to-vigorous physical activities

Socio-demographic variables included age, gender, employment status and level of education (higher vs. lower). Higher education was defined as completing tertiary education (ie college or university), and lower education was defined as everything below a tertiary education. Body mass index (BMI) was calculated by dividing self-reported weight in kilograms by the square of the self-reported height in meters, and moderate-to-vigorous physical activity in the last seven days was measured using items from the long version of the International Physical Activity Questionnaire [19].

### Statistical analyses

Inspection of the raw dataset revealed that item non-response ranged from <1 % for gender to 32 % for other leisure sitting time. Twenty imputed datasets were generated using the method of chained equations with predictive mean matching, as we assumed data were missing at random [20, 21]. Estimates from the multiple imputations were pooled using Rubin’s rules to obtain a single set of results [22]. A sensitivity analysis was performed on the original non-imputed dataset.

As the assumption of normality was not met for any of the dietary habit variables, they were dichotomized at the median for analyses. This resulted in the following categories: high (≥7 portions/week) and low fruit intake (<7 portions/week), high (≥7 portions/week) and low vegetables intake (<7 portions/week), high (≥2 glasses/week) and low sugar-sweetened beverages consumption (<2 glasses/week), high (>2 glasses/week) and low alcohol consumption (≤2 glasses/week), high (≥3 portions/week) and low sweets intake (<3 portions/week), and high (>0.5 portions/week) and low fast food intake (≤0.5 portions/week). The outcomes are defined as high consumption versus low consumption.

Two two-level (neighbourhood and individual) mixed-effects logistic regression analyses with random intercepts were first conducted for each dietary habit (=dependent variable): one model including all participants, to examine the influence of transport-related sitting time, television time, computer time and other leisure sitting time (Model 1), and another model limited to working participants, to examine the influence of work-related sitting time (Model 2). Secondly, the two-level mixed effects logistics regression analyses were repeated with addition of interaction terms of age and gender (moderators × domain-specific sedentary behaviours). Concretely, ten separate regression models were fitted for each dietary habit; five models to assess the interaction with each sedentary behaviour and age (eg transport-related sedentary behaviour × age, television time × age…), and five models to assess the interaction with each sedentary behaviour and gender (eg transport-related sedentary behaviour × gender, television time × gender…). To examine the interaction with age, age was dichotomised at 65 years, as this is generally the age of retirement, and is accompanied by a considerable change in health behaviours [23, 24]. Where there was a significant interaction effect, analyses were stratified by age group or gender. Variance inflation factors were calculated for each independent variable included in the first model, ranging from 1.07-1.15, revealing no multicollinearity [25]. All analyses were adjusted for a range of covariates, previously shown to be associated with sedentary behaviour [10, 26] or dietary habits [27–29], ie urban region, neighbourhood type, age, gender, level of education, BMI and total moderate-to-vigorous physical activity. All analyses were performed with R software, version 3.1.2 [30] and level of significance was set at a two-sided *p*-value of 0.05.

## Results

### Sample characteristics

A total of 6,037 (out of 55,893) individuals participated in the study between February and September 2014. The overall response rate was 10.8 %. The response rate for residents from low SES neighbourhoods was 9.6 %, and the response rate for residents from high SES neighbourhoods was 11.9 % [31]. Thirty-six participants were excluded from the analyses, as they did not provide information on their residential address. In total, 6,001 subjects were included in the analyses (Table 1); mean age was 51.9 years (SD = 16.4), 56.0 % of subjects were women, 53.5 % were in the higher education category, and 54.7 % were currently employed. The lowest values for minutes sitting per day were observed for transport-related sitting (mean [SD]: 1.4 [1.5] hours/day), while highest values were observed for sitting at work (4.3 [2.6] hours/day).

**Table 1:** Socio-demographic characteristics, sedentary behaviours and dietary habits of the total sample

Characteristics	Total sample (*n* = 6,001)
Socio-demographic characteristics	
Age (years), mean (SD)	51.9 (16.4)
Gender, n (%)	
Male	2612 (44.0)
Female	3329 (56.0)
Level of education, n (%)	
Lower	2505 (46.5)
Higher	2877 (53.5)
Employment status, n (%)	
Currently employed	3267 (54.7)
Currently not employed	2711 (45.3)
Household composition, n (%)	
One-person household	1219 (22.6)
Two person household	2124 (39.3)
Three-person household	848 (15.7)
Four-person household	792 (14.7)
Five-or more-person household	420 (7.7)
Domain-specific sedentary behaviours	
Transport-related sitting time (hours/day), mean (SD)	1.4 (1.5)
Work-related sitting time (hours/day), mean (SD)^a^	4.3 (2.6)
Television time (hours/day), mean (SD)	2.6 (2.1)
Computer time (hours/day), mean (SD)	1.9 (1.9)
Other leisure sitting time (hours/day), mean (SD)	1.5 (1.7)
Dietary habits	
Fruit intake (times per week), median (Q1, Q3)	7.0 (4.0, 7.0)
Vegetables intake (times per week), median (Q1, Q3)	7.0 (5.0, 7.0)
Sugar-sweetened beverages consumption (times per week), median (Q1, Q3)	2.0 (0.5, 6.0)
Alcohol consumption (times per week), median (Q1, Q3)	2.0 (0.5, 6.0)
Sweets intake (times per week), median (Q1, Q3)	3.0 (0.5, 6.0)
Fast food intake (time per week), median (Q1, Q3)	0.5 (0.5, 0.5)
Body mass index (kg/m^2^), mean (SD)	25.2 (4.5)

N for some variables is reduced due to missing data

*SD* Standard deviation, *Q1* Quartile 1, *Q3* quartile 3

^a^The mean and standard deviation of work-related sitting time was only computed for participants who were employed at the time of the survey

### Associations between domain-specific sedentary behaviours and dietary habits

Table 2 presents the main associations of domain-specific sedentary behaviours (transport-related sitting time, work-related sitting time, television time, computer time and other leisure sitting time) with dietary habits (fruit intake, vegetables intake, sugar-sweetened beverages consumption, alcohol consumption, sweets intake and fast food intake). All domain-specific sedentary behaviours were significantly associated with one or more dietary habits, except transport-related sitting time.

**Table 2:** Main effects of domain-specific sedentary behaviours on dietary habits

	Fruit intake	Vegetables intake	Sugar-sweetened beverages consumption	Alcohol consumption	Sweets intake	Fast food intake
	Adj. OR (95 % CI)	Adj. OR (95 % CI)	Adj.OR (95 % CI)	Adj. OR (95 % CI)	Adj. OR (95 % CI)	Adj. OR (95 % CI)
Model 1						
Transport-related sitting time (hours/day)	1.004 (0.994, 1.014)	1.000 (0.991, 1.010)	1.003 (0.991, 1.010)	1.000 (0.989, 1.011)	0.997 (0.987, 1.007)	0.997 (0.993, 1.002)
Television time (hours/day)	0.984 (0.976, 0.991)^***^	0.981 (0.975, 0.987)^***^	1.017 (1.010, 1.025)^***^	1.004 (0.996, 1.011)	1.005 (0.997, 1.014)	1.007 (1.003, 1.011)^***^
Computer time (hours/day)	0.992 (0.982, 0.998)^*^	0.995 (0.987, 1.002)	0.994 (0.985, 1.002)	0.988 (0.980, 0.997)^**^	0.999 (0.990, 1.008)	1.004 (1.000, 1.009)
Other leisure sitting time (hours/day)	1.010 (1.001, 1.020)^*^	1.004 (0.994, 1.014)	0.998 (0.989, 1.008)	1.014 (1.005, 1.023) ^**^	1.006 (0.997, 1.015)	1.009 (1.004, 1.013)^**^
Model 2						
Work-related sitting time (hours/day)	0.994 (0.986, 0.999)^*^	1.003 (0.996, 1.011)	0.993 (0.986, 1.000)	1.010 (1.002, 1.017)^**^	0.994 (0.987, 1.002)	0.999 (0.995, 1.003)

Outcome variables were dichotomized based on the median

All analyses were adjusted for urban region, neighbourhood type, age, educational level, gender, body mass index, and moderate-to-vigorous physical activity

*Adj. OR* adjusted odds ratio, *95 % CI* confidence interval at 95 %

^***^ *p* < 0.001, ^**^ *p* < 0.01, ^*^ *p* < 0.05

Having a higher television time was associated with four unhealthy dietary habits, namely having a lower fruit and vegetables intake, and having a higher sugar-sweetened beverages and fast food consumption. For example, per one hour increase in television time, participants have 2 % lower odds of having a high fruit intake (≥7 portions/week). Computer time was negatively associated with fruit intake and alcohol consumption. Sitting more during other leisure time activities (than watching television or using the computer) was associated with having a higher fruit, alcohol and fast food consumption. Work-related sitting time was negatively associated with fruit intake, and positively associated with alcohol consumption.

### Moderating effects of gender and age group on the association between domain-specific sedentary behaviour and dietary habits

Moderating effects of gender and age group are reported in Table 3. Gender did not moderate any of the relations between domain-specific sedentary behaviours and dietary habits. Age was only a significant moderator in the relation between other leisure sitting time and alcohol consumption. Stratified analyses by age group showed that a positive significant association between other leisure sitting time and alcohol consumption was found in adults aged below 65 years (adj. OR = 1.025, 95 % CI = 1.015,1.036), whereas no significant association was found in adults older than 65 years (adj. OR = 0.992, 95 % CI = 0.974,1.011).

**Table 3:** Interaction effects of gender and age group on the association between domain-specific sedentary behaviours and dietary intake

Outcomes:	Fruit intake		Vegetables intake		Sugar-sweetened beverages consumption		Alcohol consumption		Sweets intake		Fast food intake	
Interaction terms	Gender Adj. OR (95 % CI)	Age Adj. OR (95 % CI)	Gender Adj. OR (95 % CI)	Age Adj. OR (95 % CI)	Gender Adj. OR (95 % CI)	Age Adj. OR (95 % CI)	Gender Adj. OR (95 % CI)	Age Adj. OR (95 % CI)	Gender Adj. OR (95 % CI)	Age Adj. OR (95 % CI)	Gender Adj. OR (95 % CI)	Age Adj. OR (95 % CI)
Model 1												
Transport-related sitting time (hours/day)	0.992 (0.973, 1.012)	1.005 (0.985, 1.025)	1.003 (0.985, 1.021)	1.008 (0.987, 1.030)	0.998 (0.979, 1.017)	1.011 (0.988, 1.035)	0.999 (0.981, 1.007)	1.012 (0.990, 1.034)	0.998 (0.978, 1.018)	1.001 (0.978, 1.024)	1.004 (0.994, 1.013)	0.999 (0.989, 1.010)
Television time (hours/day)	0.992 (0.980, 1.005)	1.007 (0.994, 1.021)	1.001 (0.989, 1.013)	1.006 (0.991, 1.020)	1.005 (0.993, 1.018)	0.996 (0.982, 1.011)	0.994 (0.981, 1.018)	1.002 (0.989, 1.014)	0.994 (0.981, 1.006)	1.012 (0.997, 1.027)	1.000 (0.994, 1.007)	0.996 (0.989, 1.004)
Computer time (hours/day)	0.993 (0.978, 1.009)	1.002 (0.985, 1.018)	1.002 (0.988, 1.016)	0.997 (0.980, 1.014)	0.995 (0.981, 1.010)	1.003 (0.984, 1.021)	1.002 (0.987, 1.017)	1.015 (0.998, 1.033)	1.002 (0.988, 1.017)	1.005 (0.988, 1.023)	0.998 (0.991, 1.005)	0.999 (0.989, 1.008)
Other leisure sitting time (hours/day)	1.009 (0.993, 1.027)	1.001 (0.983, 1.020)	0.998 (0.983, 1.014)	0.993 (0.976, 1.011)	0.993 (0.976, 1.009)	1.007 (0.989, 1.025)	1.005 (0.989, 1.023)	0.983 (0.964, 0.999)^*^	0.993 (0.976, 1.009)	1.004 (0.983, 1.026)	0.999 (0.990, 1.007)	0.994 (0.985, 1.003)
Model 2												
Work-related sitting time (hours/day)	0.997 (0.986, 1.008)	1.003 (0.987, 1.020)	0.997 (0.987, 1.008)	0.995 (0.980, 1.009)	0.996 (0.985, 1.007)	0.998 (0.981, 1.015)	1.006 (0.996, 1.016)	1.000 (0.984, 1.016)	0.998 (0.988, 1.009)	1.006 (0.988, 1.024)	1.001 (0.995, 1.006)	0.999 (0.992, 1.006)

Outcome variables were dichotomized based on the median

All analyses were adjusted for urban region, neighbourhood type, age, educational level, gender, body mass index, and moderate-to-vigorous physical activity

*Adj. OR* Adjusted odds ratio, *95 % CI* confidence interval at 95 %

^*^ *p* < 0.05

## Discussion

Our first aim was to determine whether there are associations between domain-specific sedentary behaviours and dietary habits in adults. After controlling for socio-demographic factors, BMI and physical activity, eleven significant associations were found, of which nine showed that higher levels of work or leisure time (ie television time, computer time and other leisure sitting time) sedentary behaviour were associated with less healthy dietary habits. This finding may be important given that clustering of unhealthy behaviours has been found to have synergistic effects on health, which implies that the combined effects are more harmful than those from the sum of the individual unhealthy behaviours [32]. However, although several statistically significant associations were found, they do not necessarily reflect meaningful associations. Both the large sample size of this study, which may have resulted in an over rejection of the null hypothesis, and the small effect sizes, call into question the clinical relevance of the significant sedentary behaviours and dietary habits associations. In view of this, the main focus of this discussion will be on the four associations that were significant at the .001 level.

These four associations all showed that television viewing was unfavourably related to dietary habits. This is noteworthy, as television time is only one of the five domain-specific behaviours that were included in this study. More concretely, spending more time watching television was related to less frequent consumption of fruit and vegetables, and more frequent consumption of sugar-sweetened beverages and fast food. These findings confirm previously reported results [33–36], and could be explained by disrupted habituation to food cues [37, 38] or by increased exposure to unhealthy food advertisements [39, 40]. For example, Scully et al. reported that respondents were significantly more likely to eat fast foods for snacks at least once weekly if they usually watched commercial television for two or three hours/day compared to those who watched commercial television for less than two hours/day [40]. Food advertisements may not only contribute to increases in unhealthy dietary habits by promoting unhealthy food options, they may also contribute to a reduction in consumption of healthy foods, such as fruit and vegetables, through potentially misleading messages about the nutritional value of food items [41]. Furthermore, individual-level factors, such as attitudes and norms about healthy lifestyles, or a number of other aspects affecting motivation, may also partially explain these results. For example, results from Mata et al. [42] suggest the existence of specific motivational “spill-over” effects across health behaviours during lifestyle change. The many associations between television time and unhealthy dietary habits seem likely to explain, at least in part, the adverse impact of television time on obesity and related chronic diseases [43–45], and thus clarify the stronger negative influence of television time on health compared to total sedentary behaviour. In this way, results of this study also contribute to resolve the controversy surrounding the direct (ie the action of sitting in front of the television leads to obesity) or indirect (ie sitting in front of the television leads to obesity via dietary habits) influence of television time on obesity and related chronic diseases, by suggesting that the effects are rather indirect.

In contrast, associations with other sedentary behaviours are weaker, less clinically relevant, and less consistent. For example, computer time was related to both unhealthy and healthy dietary habits: spending more computer time was related to both eating fruit and drinking alcohol less frequently. Given the considerable difference between television and computer time on dietary habits, our findings support the importance of analysing these two behaviours separately, rather than using a summary construct such as ‘screen time’, used in some previous papers (eg [46–48]). Other leisure sitting time was associated with two unhealthy dietary habits: more frequent consumption of both alcohol and fast food. However, the association with alcohol consumption was only significant in adults aged below 65 years, which is consistent with the fact that alcohol consumption decreases with increasing age [49]. As far as we know, there are no studies on the association of other types of leisure sitting time on dietary habits. However, given that other leisure time activities form a heterogeneous grouping, including visiting restaurants, socializing with friends, or going to a pub, the positive association with alcohol consumption and fast food intake could be expected. Nevertheless, more research is needed to confirm these results. Finally, spending more time sitting for work was positively related to alcohol consumption and negatively related to fruit intake. A cross-sectional Australian study found that those who sat longer at work had greater psychological distress [50], itself plausibly associated with higher alcohol consumption [51]. However, evidence is lacking on both the direction of causality and generalizability. On the other hand, evidence of an association between sedentary behaviour and fruit intake has been conflicting. Contrary to our findings, Pereira et al. showed that higher sitting time at work was associated with higher fruit intake [52]. The reason for these inconsistent results is unclear; however, it might be that other characteristics, such as type of work, may have influenced the relationship.

The second aim of this study was to assess whether any association differed by age and gender, as previous studies showed mixed results concerning the moderating role of age and gender [7]. Our results did not provide evidence to support a potential moderating role of age or gender, given that only one significant moderating effect was found, ie age moderated the association between leisure sitting time and alcohol consumption. Consequently, most associations between domain-specific sedentary behaviour and dietary habits seem to be consistent across men and women, and across adults and older adults.

The main strength of this study was its innovative nature, as this study was the first to link different domain-specific sedentary behaviours to dietary habits. Examining domain-specific sedentary behaviour is important, as specific associations may be masked when analysing total sedentary behaviour. A second strength was the large sample size, which ensures adequate power to identify associations. A final strength was that the study sample was recruited from five urban regions in different European countries, which increases the external validity of our findings to Europe.

Despite these strengths, some limitations affect the validity and generalizability of the results. Firstly, information on both domain-specific sedentary behaviours and dietary habits was self-reported and therefore prone to social desirability and recall biases. Moreover, dietary habits were measured using single items, which may have reduced the accuracy. As both the predictor variables, the outcomes and the confounders were self-reported, observed associations may be the result of correlated error. Future studies should use more precise dietary assessment with quantitative assessment of dietary intake, preferably in combination with objective measurement instruments for sedentary behaviour. These objective measurement instruments (eg accelerometers, or inclinometers [53]) should be combined with Global Positioning Systems and/or diaries to gain insight into domain-specific sedentary behaviours. Secondly, the cross-sectional study design does not allow for causal inferences between domain-specific sedentary behaviours and dietary habits. Finally, despite sending reminders, the response rate was relatively low, which may have resulted in a selection bias. Although there is a good representation of men (44 %) and women (56 %), lower (46.4 %) and higher (53.6 %) educated individuals as well as younger (from age 18 years) and older (up to age 109 years) adults [31], it remains likely that generally more healthy people participated, suggesting that we may have underestimated domain-specific sedentary behavior and unhealthy dietary habits. Possible reasons for the low response rate include first the oversampling of low SES residents. Low SES residents have been shown to be less likely to participate in a health survey [17]. However, as we aimed to have a heterogeneous sample with as many low SES residents as high SES residents, we decided to oversample the former, which is likely to have led to a lower overall response rate. Secondly, with regard to the absence of an upper age limit, we know that there may be attrition in surveys where older people are less likely to be able to complete a survey due to, for example, limited cognitive function, or vision impairment [54]. In addition, the questionnaire was mainly administered online. Previous studies have indicated that Internet use drops off significantly after the age of 75 [55], also potentially contributing to a lower response rate. Thirdly, the survey was relatively long. Participants spend on average 25.1 ± 12.4 min to complete the questionnaire, which contained 50 key questions on 30 pages. Finally, we recognize that, in an era of frequent opinion polls and market research, people may react to what they perceive as over-surveying (i.e. become fed up with surveys). Although each of these factors, on their own, may not have had a large impact, they all act to reduce the response rate so, in combination, the effect may be appreciable.

## Conclusion

Domain-specific sitting behaviours are only weakly related to unhealthy dietary behaviours. This suggests that individual level behavioural interventions focusing on sedentary behaviour will not be sufficient to improve dietary habits. Nevertheless, large-scale multi-level interventions, affecting both individual and environmental factors, are required. If future intervention designers, however do decide to develop an individual behavioural intervention to prevent obesity and related chronic diseases, television time should be recommended as main target, as television time was the only domain of sedentary behaviour that was consistently related to unhealthy dietary habits. Our results also suggest a limited moderating role of age and no moderating role of gender in the association between domain-specific sedentary behaviour and dietary habits. The fact that almost none of the associations were moderated by age or gender suggests that these associations, and possibly also the effects of interventions targeting both behaviours, may largely hold across age and gender groups. However, more research is needed in adult populations to confirm the lack of moderating effects of socio-demographic factors on the association between domain-specific sedentary behaviour and dietary habits, as this was the first study to include interactions with age and gender in an adult population.

## Abbreviations

95 % CI: Confidence interval at 95 %
Adj. OR: Adjusted odds ratio
BMI: Body mass index
SES: Socioeconomic status
